# The Electrocardiogram of an ER Patient With Chest Pain

**DOI:** 10.4021/cr9w

**Published:** 2011-01-20

**Authors:** Arunkumar Panneerselvam, Rajiv Ananthakrishna, Prabhavathi Bhat, Manjunath C Nanjappa

**Affiliations:** a Department of Cardiology, Sri Jayadeva Institute of Cardiovascular Sciences & Research, Bangalore, India

**Keywords:** Myocardial infarction, Atrial arrhythmias, Electrocardiogram

## Abstract

The electrocardiogram (ECG) is an essential investigation in the evaluation of chest pain in the emergency room (ER). Correct interpretation of the ECG findings, determines the diagnosis and management strategy. This ECG spot diagnosis will improve the skills of the residents and physicians working in ER.

## Case Report

**Figure 1:**
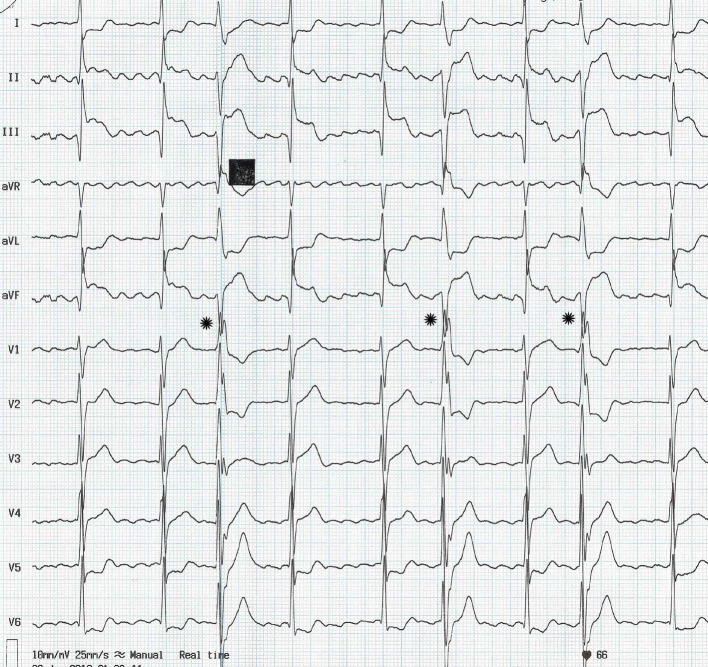
The electrocardiogram recorded in emergency room (ER).

A 46-year-old man, who was earlier in sinus rhythm, presented with anginal type of chest pain of three hours duration. The electrocardiogram recorded in emergency room (ER) is shown in Figure 1.

### ECG interpretation

The electrocardiogram shows ST segment elevation in inferior leads suggesting inferior wall myocardial infarction. The underlying rhythm is atrial fibrillation. The irregularly, irregular R-R interval coupled with the continuously varying morphology of the fibrillatory waves establishes the diagnosis of atrial fibrillation. Though the rhythm appears like atrial flutter, this is unlikely due to the above mentioned reasons. Atrial flutter is characterized by undulating sawtooth like monomorphic flutter waves and the R-R interval is regularly, irregular due to fixed heart block. The varying atrioventricular block results in long-short cycle sequences that are followed by QRS complexes with increased width (130 msec) (indicated by * in Figure 1).. These wide QRS complexes could be supraventricular beats with aberrant conduction i.e. Ashman’s phenomenon or ventricular premature beats (VPB). The long-short cycle sequence predisposes to both aberrant conduction as well as VPB. The absence of longer returning cycle following the wide QRS beats points to probable diagnosis of aberrancy [1]. But, the relatively fixed coupling interval of 640 - 680 msec [1], reversal of the “rabbit ears” in the wide QRS beats in V1 (RSr’ instead of rSR’) [2] and the R/S ratio of < 1 in V6 [3] argue against the diagnosis of Ashman’s phenomenon.

### Diagnosis

Atrial fibrillation complicating inferior wall myocardial infarction with intermittent ventricular premature beats.

### Clinical course

As the fast, irregular ventricular response aggravates myocardial ischemia, reverting to sinus rhythm is mandatory. Since it is a new onset atrial fibrillation, electrical cardioversion can be done safely. The patient also requires reperfusion of the infarct related coronary artery at the earliest. He underwent successful fibrinolysis and electrical cardioversion to sinus rhythm. Post cardioversion he was started on oral beta-blocker therapy. The further in hospital course was uneventful and he continued to be in sinus rhythm at 4 months of follow-up.

## Discussion

Atrial fibrillation complicating acute myocardial infarction is associated with increased morbidity and mortality. The proposed mechanism of atrial fibrillation in acute myocardial infarction includes congestive heart failure, ischemia and infarction of the conduction system, atrial infarction or ischemia, and vagal reflexes or increased sympathetic activity [4]. Electrical cardioversion is the treatment of choice to revert to sinus rhythm and beta-blockers should be an essential part of the treatment regimen.

In conclusion, electrocardiographic recognition of atrial arrhythmias is important to risk stratify acute coronary syndrome patients. Identification and prompt treatment of atrial arrhythmias improve outcomes.
